# Does the efficacy of neurodynamic treatments depend on the presence and type of criteria used to define neural mechanosensitivity in spinally-referred leg pain? A systematic review and meta-analysis

**DOI:** 10.4102/sajp.v78i1.1627

**Published:** 2022-07-22

**Authors:** Tawanda Murape, Timothy R. Ainslie, Cato A. Basson, Annina B. Schmid

**Affiliations:** 1 Department of Sport, Health Sciences and Social Work, Faculty of Health and Life Sciences, Oxford Brookes University, Oxford, United Kingdom; 2 Department of Physiotherapy, Faculty of Health Sciences, University of the Witwatersrand, Johannesburg, South Africa; 3 Nuffield Department of Clinical Neurosciences, Faculty of Medical Sciences, University of Oxford, Oxford, United Kingdom

**Keywords:** spinally referred leg pain, sciatica, neurodynamics, neural mobilisation, straight leg raise, slump, nerve-related pain

## Abstract

**Background:**

It remains unclear whether definite neural mechanosensitivity (NM) is required for neural mobilisations to be beneficial in people with spinally referred leg pain.

**Objective:**

To determine whether the efficacy of neural mobilisations in patients with spinally referred leg pain depends on the presence and type of criteria used to define NM.

**Method:**

PubMed, CINAHL, Cochrane Central Register of Controlled Trials, PEDro and Science Direct were searched from 1980 to March 2020. Randomised controlled trials evaluating the efficacy of neural mobilisations on pain and disability in spinally referred leg pain were included. Studies were grouped according to the certainty of NM into NM_definite_, NM_unclear_, NM_untested_ and NM_absent_. Effects on pain and disability and subgroup differences were examined.

**Results:**

We identified 21 studies in 914 patients (3 NM_definite_, 16 NM_unclear_, 2 NM_untested_, 0 NM_absent_). Meta-analysis revealed medium to large effect sizes on pain for neurodynamic compared to control interventions in NM_definite_ and NM_unclear_ groups. For disability, neurodynamic interventions had medium to large effects in NM_unclear_ but not NM_definite_ groups. NM_untested_ studies could not be pooled.

**Conclusion:**

The nonexistence of studies in patients with negative neurodynamic tests prevents inferences whether neural mobilisations are effective in the absence of NM. The criteria used to define NM may not impact substantially on the efficacy of neural mobilisations. The mostly high risk of bias and heterogeneity prevents firm conclusions.

**Clinical implications:**

Neural mobilisations seem beneficial to reduce pain and disability in spinally referred leg pain independent of the criteria used to interpret neurodynamic tests.

## Introduction

Spinally referred leg pain is a common variation of lower back pain with a prevalence of up to 43% (Konstantinou & Dunn 2008). Recent systematic reviews have demonstrated that neural mobilisations are effective in reducing the pain and disability for people with spinally referred leg pain (Basson et al. 2017; Neto et al. 2017). Neural mobilisations use active and passive movements designed to facilitate movement or tensioning of neural tissue in relation to their surrounding structures (the interface). In early publications, neural mobilisations were recommended to specifically address patient presentations involving neural mechanosensitivity (NM) (Elvey 1979, 1997). Neural mechanosensitivity is clinically identified through heightened sensitivity of peripheral nerve trunks to pressure or tension (Butler 2000). Neurodynamic tests such as the straight leg raise (SLR) and slump test have been developed to elongate the nerve bed, consequently increasing strain on neural structures (Butler 2000). Currently recommended criteria for a positive neurodynamic test and thus heightened NM include at least partial reproduction of patients’ symptoms plus their modification with structural differentiation using movement at a site remote to the painful area to further load or unload the nervous system (Nee et al. 2012).

Despite these early recommendations, it remains unclear whether neural mobilisations are indeed only beneficial for patients with confirmed NM or also for patients with spinally referred pain without clear signs of NM. Undeniably, spinally referred leg pain comprises a heterogeneous group of patients (Schmid & Tampin 2018), including those with NM, but also those with predominant somatic referred pain, radicular pain or radiculopathy. The absence of a diagnostic gold standard and agreed-upon taxonomy means that inconsistent diagnostic and eligibility criteria are used in studies (Lin et al. 1994). Whereas tests for NM are often used to include patients in trials of conservative care for spinally referred leg pain, other selection criteria such as pain distribution, neurological testing and imaging are also common (Lin et al. 2014). This divergence of diagnostic criteria is likely to result in the inclusion of distinct patient populations under the same terminology of spinally referred leg pain. Preclinical literature suggests that neurodynamic treatment may have beneficial effects beyond reducing NM, including increased nerve regeneration, increased muscle strength or decreased neuroinflammation (Da Silva et al. 2015; Giardini et al. 2017; Santos et al. 2014). Knowing whether neural mobilisations are only beneficial in patients with NM or also in those without NM would help clinicians guide their management. The objective of our systematic review was therefore to determine the extent to which the criteria used to define mechanosensitivity, influence the treatment outcomes of neural mobilisation interventions on pain and disability in patients with spinally referred leg pain.

## Methods

Our review is reported according to the updated guidance for the Preferred Reporting Items for Systematic Reviews and Meta-Analyses (Page et al. 2021). The protocol of the study was not prospectively registered, but can be downloaded from https://doi.org/10.5287/bodleian:dm74JX0Bm.

### Literature search

We built our search on a systematic review previously published by Basson et al. (2017) and included the randomised controlled trials evaluating the efficacy of neural mobilisations in populations with spinally referred leg pain identified in that review (search performed in January 2016). We also performed a new search from 01 January 2016 to 26 March 2020 to identify newly published randomised controlled trials of neural mobilisation in this patient population. The databases searched were PubMed (Medline), CINAHL, Cochrane Central Register of Controlled Trials, PEDro and Science Direct. The main search terms related to randomised controlled trials, spinally referred leg pain and neural mobilisation (see Online Appendix 1 for details of the searches). Electronic searches were supplemented by hand-searching of reference lists of relevant articles and previous systematic reviews. The search was limited to studies written in English and those which included human participants.

### Selection process and eligibility criteria

Study selection of the new search was completed by a single reviewer in two stages. Firstly, study titles and abstracts were screened for eligibility. Secondly, the full paper was obtained of those papers passing the title and abstract screen, and a comprehensive assessment for eligibility was performed. The following eligibility criteria were used: randomised controlled trials, published in English, which evaluated the effect of neural mobilisation in participants with spinally referred leg pain over the age of 18 compared to a control intervention which did not include neural mobilisation (e.g., sham neural mobilisation, other intervention or no intervention). Neural mobilisation could be achieved through exercises or manual techniques aimed at the mobilisation of neural tissue or the neural interface. Studies had to report outcome measures related to pain severity (e.g., numerical pain rating scale or visual analogue scale [VAS]) and/or disability (e.g., Oswestry Disability Index [ODI]). Case reports, case-control and cohort studies as well as studies on animals or healthy participants were excluded.

### Risk of bias assessment

Papers which met the inclusion criteria were assessed for risk of bias by two independent reviewers (T.M., T.A.) using the Cochrane risk-of-bias tool (Higgins et al. 2011). Agreement rates are reported, and discrepancies were resolved through discussion with a third reviewer (A.B.).

### Data extraction

Data were extracted by a single reviewer (T.M.) from eligible studies and double-checked by a second investigator (A.S.). For all studies, we extracted data on the number of patients in each group, patient demographics, type of neural mobilisation and control interventions, timing of assessment, type of outcome measures and results (e.g., mean and standard deviations). In the case of unclear study information, authors were contacted to obtain the required information.

In addition, we extracted the criteria used to define the patient population and in particular whether studies included patients with established NM or not. Studies were grouped according to the certainty of the presence of NM as follows. The NM_definite_ subgroup consisted of studies which used tests for NM as an essential inclusion criterion and adhered to recommended principles of (1) at least a partial symptom reproduction plus (2) modulation of symptoms upon structural differentiation (sensitising movements at a site distant to the symptoms) (Nee et al. 2012). The NM_unclear_ subgroup consisted of studies which used tests for NM as an essential inclusion criterion but did either not specify which principles were used to deem a test positive or the principles used did not conform with current recommendations (e.g., range of motion deficit upon SLR, symptom reproduction without mention of structural differentiation). The NM_untested_ subgroup consisted of studies which did not include tests for NM as part of their inclusion criteria. If studies performed neurodynamic tests as part of their inclusion criteria, but only included patients with negative neurodynamic tests, these studies were allocated to the NM_absent_ subgroup.

### Data synthesis and meta-analysis

Data are reported separately for studies that included patients in the NM_definite_, NM_unclear_, NM_untested_ and NM_absent_ subgroups. Where more than two studies reported the same outcome measures, data from the final follow-up time point were pooled in a statistical meta-analysis and presented as forest plots using the computer software Cochrane Review Manager (The Cochrane Collaboration 2020). Means, standard deviations and sample sizes from studies reporting continuous data were used to calculate standardised mean differences (SMD) and 95% confidence intervals (CI). Separate random effects models and inverse variance weighting methods were used to compare effects on pain and disability between neural mobilisation and control groups. Subgroup differences were compared using Chi-square tests for heterogeneity (Cochran’s Q test). Heterogeneity was defined with I^2^ statistics and interpreted as ‘might not be important’ (0%–40%), ‘moderate’ (30%–60%), ‘substantial’ (50%–90%), and ‘considerable’ (75%–100%) (Higgins et al. 2011).

### Ethical considerations

Our review followed all ethical standards for research without direct contact with human or animal subjects.

## Results

### Study selection

**FIGURE 1:**
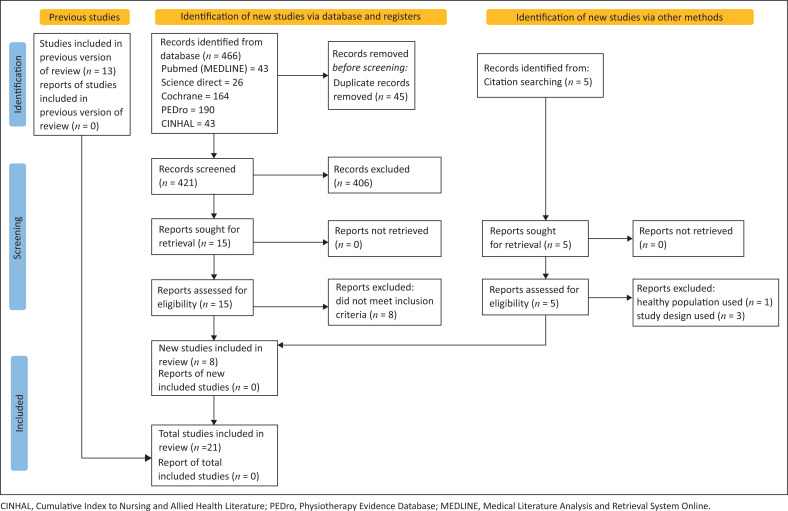
Preferred Reporting Items for Systematic Reviews and Meta-Analyses (PRISMA) flowchart of study selection.

Our previous search from January 1980 to January 2016 identified 13 studies to be included (Basson et al. 2017). The new search from 2016 onwards identified a total of 291 studies, of which eight were deemed eligible. This resulted in a total of 21 studies included in our systematic review (Figure 1).

### Quality assessment

**FIGURE 2:**
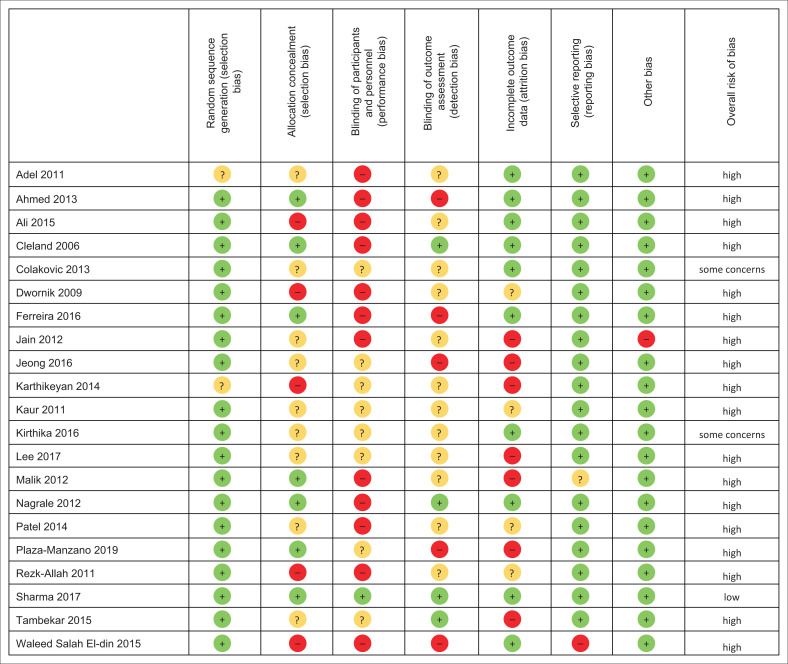
Risk of bias of included studies.

Risk-of-bias assessment revealed that 18 out of 21 studies were classified as high risk of bias (Figure 2). In addition to the lack of blinding of participants and personnel (52.4% of studies), blinding of outcome assessment (19%) and incomplete outcome data (33.3%) were the most frequently identified high-risk biases. Investigator agreement for the Cochrane risk-of-bias tool was 86.4%.

### Characteristics of included studies

Characteristics of the 21 studies are summarised in Online Appendix 2, Table-A2, which also displays to which mechanosensitivity subgroup each study belongs. In total, *n* = 914 participants were included. The smallest sample size was *n* = 22 (Lee & Kim 2017) and the largest *n* = 108 (Dwornik et al. 2009).

All studies monitored pain and/or disability either as primary or secondary outcome measures. The VAS and numeric pain rating scale (NPRS) were the most commonly used outcome measures to monitor pain. To quantify disability, most studies used a version of the Oswestry Disability Index (MODI or ODI).

The type of neural mobilisation technique and dosage varied amongst groups. The techniques used included slump (Ali et al. 2015; Cleland et al. 2006; Jain et al. 2012; Jeong et al. 2016; Karthikeyan, Jothikaran & Kiran 2014; Kirthika et al. 2016; Malik, Kataria & Sachdev 2012; Nagrale et al. 2012; Patel 2014; Rezk-Allah, Shehata & Gharib 2011; Tambekar et al. 2015; Waleed 2015), SLR (Adel 2011; Ahmed et al. 2013; Kaur & Sharma 2011; Malik et al. 2012; Rezk-Allah et al. 2011; Waleed 2015), bent leg raise mobilisation (Dwornik et al. 2009; Patel 2014; Tambekar et al. 2015) and neural mobilisations in side lying (Colakovic & Avdic 2013; Ferreira et al. 2016; Lee & Kim 2017). One study reported a neural slider technique of the sciatic nerve involving the hip, knee and foot (Plaza-Manzano et al. 2020). Another study did not describe their neural mobilisation intervention (Sharma & Sheth 2017). More detailed information on the techniques and dosages used can be found in Online Appendix 2, Table 1-A2.

The neural mobilisation interventions were a standalone treatment in six studies (Dwornik et al. 2009; Kaur & Sharma 2011; Lee & Kim 2017; Patel 2014; Tambekar et al. 2015; Waleed 2015) and were combined with exercise programmes in 15 studies (Adel 2011; Ahmed et al. 2013; Ali et al. 2015; Cleland et al. 2006; Colakovic & Avdic 2013; Ferreira et al. 2016; Jain et al. 2012; Jeong et al. 2016; Karthikeyan et al. 2014; Kirthika et al. 2016; Malik et al. 2012; Nagrale et al. 2012; Plaza-Manzano et al. 2020; Rezk-Allah et al. 2011; Sharma & Sheth 2017). The control groups were diverse, including lumbar stabilisation, lumbar mobility exercises, hamstring stretching, advice, physical modalities or a combination treatment.

### Mechanosensitivity subgroups

Three studies (Ferreira et al. 2016; Kaur & Sharma 2011; Sharma & Sheth 2017) were categorised to the NM_definite_ subgroup. Sixteen studies (Adel 2011; Ahmed et al. 2013; Ali et al. 2015; Cleland et al. 2006; Colakovic & Avdic 2013; Dwornik et al. 2009; Jain et al. 2012; Jeong et al. 2016; Karthikeyan et al. 2014; Kirthika et al. 2016; Lee & Kim 2017; Malik et al. 2012; Nagrale et al. 2012; Patel 2014; Plaza-Manzano et al. 2020; Tambekar et al. 2015) were attributed to the NM_unclear_ subgroup. Of those, eight studies (Ahmed et al. 2013; Colakovic & Avdic 2013; Jeong et al. 2016; Karthikeyan et al. 2014; Malik et al. 2012; Patel 2014; Plaza-Manzano et al. 2020; Tambekar et al. 2015) used pain provocation within a certain range of motion (15–75 degrees hip flexion) as a criterion for NM. Three studies (Adel 2011; Cleland et al. 2006; Nagrale et al. 2012) used pain provocation as a criterion without reporting whether structural differentiation was applied. One study (Jain et al. 2012) used either symptom reproduction during slump testing or symptom decrease during cervical extension. Three studies (Dwornik et al. 2009; Kirthika et al. 2016; Lee & Kim 2017) did not specify the criteria used during neurodynamic testing. Two studies (Rezk-Allah et al. 2011; Waleed 2015) were attributed to the NM_untested_ subgroup. Their inclusion criteria did not evaluate NM, but instead used abnormal electromyography and prolonged latency of H- reflex >30 ms (no further specification of which nerve or test criteria) (Rezk-Allah et al. 2011) magnetic resonance imaging confirming lumbar disc herniation at L5-S1 disc level (Waleed 2015). No study was identified for the NM_absent_ subgroup.

#### NM_definite_ subgroup

Participants from three studies were identified as NM_definite_ (total sample size = 111) (Ferreira et al. 2016; Kaur & Sharma 2011; Sharma & Sheth 2017). Ferreira et al. (2016) investigated a nerve slider technique with advice to remain active compared to a control group which only received advice to remain active. At the 2-week follow-up, no significant between-group difference was present, whilst the neural mobilisation group had significantly improved leg pain compared to the control group at the 4-week follow-up. Disability did not differ significantly between groups at any time point. Kaur and Sharma (2011) investigated neural mobilisation using passive SLR mobilisation in comparison to an exercise programme consisting of back mobilisation exercises (e.g., pelvic tilting, prone back extension). The results showed a greater improvement in pain and reduction in disability in the neural mobilisation group than in the exercise group at 10 days follow-up. Sharma and Sheth (2017) used a remote or local slider and tensioner neural mobilisation technique which was adjusted based on the location of symptoms and compared it to ‘conventional’ treatment consisting of hot packs, stabilisation and core exercises. Following a 7-day period, there was a significant between-group difference favouring neural mobilisation for disability and pain during activity. Pain at rest was not different between groups.

**FIGURE 3:**
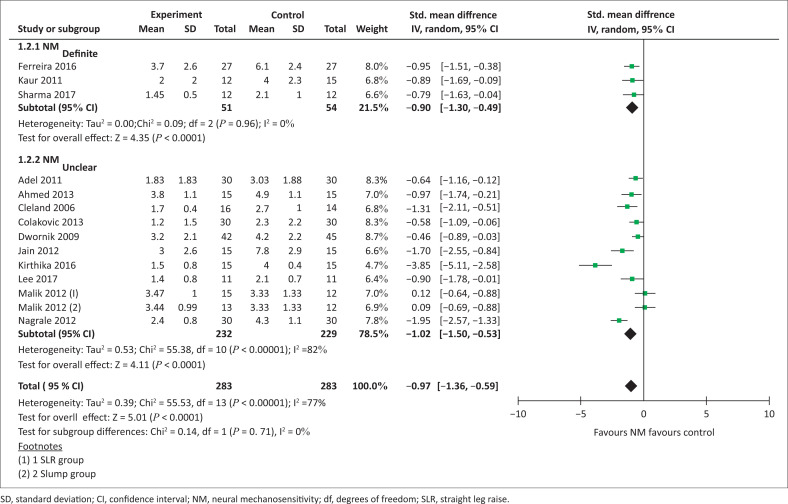
Meta-analysis for pain in people with spinally referred leg pain.

**Meta-analysis for pain (NM_definite_):** All three studies could be included in the meta-analysis for pain (VAS) in the NM_definite_ subgroup (total sample size = 105) (Ferreira et al. 2016; Kaur & Sharma 2011; Sharma & Sheth 2017). Pooling showed a significant effect favouring neural mobilisation over control interventions (standardised mean difference [SMD] −0.90 [95% CI −1.30– −0.49], *p* < 0.0001, Figure 3). Heterogeneity was considered not important (I² = 0%).

**FIGURE 4:**
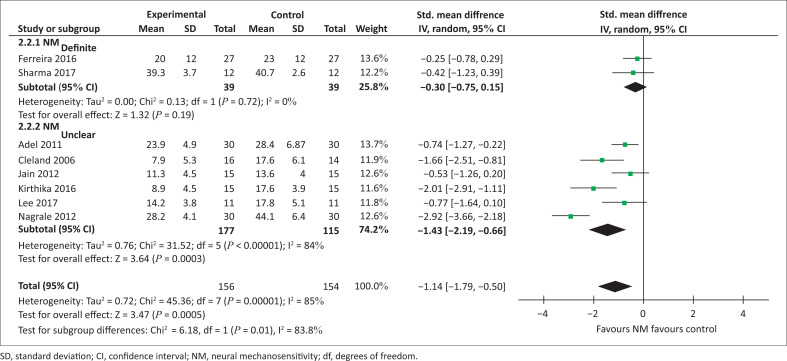
Meta-analysis for disability in people with spinally referred leg pain.

**Meta-analysis for disability (NM_definite_):** Two studies were included in the meta-analysis for disability (MODI) in the NM_definite_ subgroup (total sample size = 78) (Ferreira et al. 2016; Sharma & Sheth 2017). Pooling did not identify between-group differences (SMD −0.30 [95% CI −0.75–0.15], *p* = 0.19, Figure 4). Heterogeneity between studies was considered not important I² = 0%)

#### NM_unclear_ subgroup

In 16 studies, the presence of NM remained unclear (total sample size = 693) (Adel 2011; Ahmed et al. 2013; Ali et al. 2015; Cleland et al. 2006; Colakovic & Avdic 2013; Dwornik et al. 2009; Jain et al. 2012; Jeong et al. 2016; Karthikeyan et al. 2014; Kirthika et al. 2016; Lee & Kim 2017; Malik et al. 2012; Nagrale et al. 2012; Patel 2014; Plaza-Manzano et al. 2020; Tambekar et al. 2015). Of these, seven studies (Ali et al. 2015; Cleland et al. 2006; Dwornik et al. 2009; Jain et al. 2012; Jeong et al. 2016; Kirthika et al. 2016; Nagrale et al. 2012) provided neural mobilisation in a slump position and reported greater improvements in pain and disability for neural mobilisation compared to control interventions. Of the remaining studies, Ahmed et al. (2013) compared an SLR neural mobilisation plus conventional physiotherapy (spinal flexion or extension exercises and transcutaneous electrical nerve stimulation) to conventional physiotherapy alone. The findings revealed a significant between-group difference for pain and disability favouring the SLR neural mobilisation plus conventional physiotherapy group. Colakovic and Avdic (2013) compared lumbar stabilisation exercises plus oscillating neural mobilisation in side-lying to lumbar stabilisation exercises and active range of movement exercises for back and distal extremities. The findings revealed a significant between-group difference for pain and SLR range of motion favouring neural mobilisation. Lee and Kim (2017) compared a neural slider technique and physiotherapy (including superficial thermal treatment and interference wave) to hamstring stretching and physiotherapy. Pain alleviation was more pronounced with neural mobilisation. Plaza-Manzano et al. (2020) compared neural slider techniques and motor control exercises (consisting of bridging and four-point kneeling) to motor control exercises alone. There was no significant between-group difference for pain and disability. Tambekar et al. (2015) compared two neural mobilisation techniques, Mulligan’s bent leg raise to Butler’s neural tissue mobilisation. Both reduced the pain immediately post-treatment, but this was not sustained at the 24-h follow-up and no between-group differences were apparent. Patel (2014) compared slump stretch to Mulligan’s bent leg raise and reported no significant between-group differences for pain. Adel (2011) compared a SLR stretch plus lumbar stabilisation exercises and a standardised exercise programme to lumbar stabilisation exercises and standardised exercise programme (Adel 2011). The findings revealed a significant between-group difference post intervention for pain and disability favouring the SLR stretch. Malik et al. (2012) compared lumbar stabilisation alone to SLR mobilisation plus lumbar stabilisation exercises alongside slump neural mobilisation plus lumbar stabilisation exercises. The findings revealed that SLR and slump mobilisations are equally effective in reducing pain. Karthikeyan et al. (2014) compared joint mobilisation plus static spinal exercise to slump mobilisation. The study concluded both interventions to be beneficial for pain and disability but no significant between-group differences were reported.

**Meta-analysis for pain (NM_unclear_):** Ten studies (Adel 2011; Ahmed et al. 2013; Cleland et al. 2006; Colakovic & Avdic 2013; Dwornik et al. 2009; Jain et al. 2012; Kirthika et al. 2016; Lee & Kim 2017; Malik et al. 2012; Nagrale et al. 2012) were included in the meta-analysis for pain in the NM_unclear_ subgroup (total sample size = 461). As Malik et al. (2012) compared both a SLR and slump mobilisation group to control intervention, these data are reported separately. Pooling showed a significant effect favouring neural mobilisation compared to control interventions consisting of either exercise or lumbar mobilisation and exercises (SMD −1.02 [95% CI −1.50– −0.53], *p* < 0.0001, Figure 3). Heterogeneity was substantial to considerable (I² = 82%).

**Meta-analysis for disability (NM_unclear_):** Six studies (Adel 2011; Cleland et al. 2006; Jain et al. 2012; Kirthika et al. 2016; Lee & Kim 2017; Nagrale et al. 2012) were included in the meta-analysis for disability in the NM_unclear_ subgroup (total sample size = 232). Pooling revealed a significant difference favouring neural mobilisation over control interventions consisting of lumbar stabilisation (Jain et al. 2012; Nagrale et al. 2012), standardised exercise programme (including squats, pelvic tilts and bridging) (Cleland et al. 2006; Kaur & Sharma 2011; Kirthika et al. 2016) and hamstring stretching (Lee & Kim 2017) (SMD −1.43 [95% CI −2.19– −-0.66], *p* = 0.0003, Figure 4). Heterogeneity was substantial to considerable (I² = 84%).

#### NM_untested_ subgroup

In two studies, the presence of neural mechanosensitivity remained untested (Rezk-Allah et al. 2011; Waleed 2015). A meta-analysis could not be performed because the authors compared two different neural mobilisation exercises without including a non-neural mobilisation control group. Rezk-Allah et al. (2011) applied a slump mobilisation in comparison to a SLR mobilisation; a significant reduction in pain in both groups was reported with no significant between-group differences. Waleed (2015) applied slump mobilisation plus SLR in comparison to lumbar manipulation plus rotation with SLR. A more pronounced decrease of pain and disability was reported in the group which received the lumbar manipulation.

### Subgroup comparison

Tests for subgroup differences (NM_definite_ vs. NM_unclear_) revealed comparable benefits on pain independent of the criteria used to define NM (Chi^2^ = 0.14, *p* = 0.71, I^2^ = 0%). For disability, effects were larger for the NM_unclear_ than the NM_definite_ subgroup; however, heterogeneity was substantial to considerable (chi^2^ = 6.18, *p* = 0.01, I^2^ = 83.8%).

## Discussion

Our review identified 21 studies evaluating neural mobilisation interventions in *n* = 914 people with spinally referred leg pain. No study included patients with negative neurodynamic tests in their study population, which prevents any inferences on whether neural mobilisations are effective even in the absence of NM. Only three studies described the criteria used to define a neurodynamic test as positive in sufficient detail such that their patients could be classified as displaying definite NM. Two studies did not use neurodynamic tests as an inclusion criterion, and in 16 studies, it remained unclear whether patients had definite NM because of either insufficient information provided or criteria that did not allow firm conclusions. The meta-analysis suggested medium to large effect sizes of neural mobilisation interventions compared to control treatment on measures of pain, irrespective of the criteria used to determine nerve mechanosensitivity. For disability, meta-analysis demonstrated medium to large effects of neural mobilisation compared to control treatments for patients with NM_unclear_ but not NM_definite_. The mostly high risk of bias of included studies, small numbers of studies in the NM_definite_ subgroup and high heterogeneity of studies in the NM_unclear_ subgroup limit firm conclusions. Nevertheless, our findings currently do not support the view that the criteria used to define NM of the lower extremity may impact substantially on the clinical efficacy of neural mobilisations.

Our review clearly highlights the challenges associated with the lack of a diagnostic reference standard for spinally referred leg pain. Similar to previous reports, a wide range of inclusion criteria and their combination were used amongst studies (e.g., symptom localisation, magnetic resonance imaging findings, NM) (Lin et al. 2014). Most studies identified in our review (19 out of 21) included mechanosensitivity testing as part of their inclusion criteria. However, only three (Ferreira et al. 2016; Kaur & Sharma 2011; Sharma & Sheth 2017) adhered to the recommended criteria to determine the outcome of nerve mechanosensitivity tests (partial symptom reproduction plus structural differentiation) (Nee et al. 2012). These recommendations were originally based on data from upper limb neurodynamic tests rather than SLR or slump, which were used in our studies. Nevertheless, structural differentiation and confirmatory manoeuvres have been part of the early publications of the Lasègue sign (Forst 1969) as well as slump test (Maitland 1985). The face validity of structural differentiation is strongly backed up in the upper extremity with biomechanical (Coppieters & Butler 2008; Coppieters, Hough & Dilley 2009) as well as experimental pain studies (Coppieters, Alshami & Hodges 2006). In the lower extremity, most studies similarly show altered peripheral nerve movement during neurodynamic testing, including differentiation at distant sites (Sierra-Silvestre et al. 2018) whilst one study did not (Ellis et al. 2017).

Nevertheless, and as highlighted in our review, different authors used a wide range of criteria for lower limb neurodynamic tests, including symptom provocation (Ali et al. 2015; Cleland et al. 2006; Nagrale et al. 2012) and range of motion (Jeong et al. 2016; Patel 2014; Plaza-Manzano et al. 2020; Tambekar et al. 2015). Unfamiliar symptoms are provoked during a SLR even in the healthy population (mean 39.6 ± 13.7 degrees hip flexion) and can be altered with sensitising movements (Boyd et al. 2009). Hence, reliance on range-of-motion cut-offs in isolation to interpret neurodynamic tests would require lower hip angles than used in the included studies, e.g., <45 degrees (Colakovic & Avdic 2013), 30–70 degrees (Jeong et al. 2016), 35–70 degrees (Tambekar et al. 2015) or 40–70 degrees (Plaza-Manzano et al. 2020). Overall, our findings do not support the view that the criteria used to determine the outcome of neurodynamic tests have a major impact on the efficacy of neurodynamic interventions for people with spinally referred leg pain. Indeed, our meta-analyses confirmed that outcomes for pain improved comparably in both NM_definite_ and NM_unclear_ subgroups. For disability, neural mobilisation interventions were superior to control interventions in the NM_unclear_ but not the NM_definite_ subgroup. The subgroup comparison indicated that the NM_unclear_ group may outperform the NM_definite_ subgroup. Whereas these findings are intriguing, they must be interpreted in the light of high risk of bias, the small number of studies and participants included in the NM_definite_ subgroup and considerable heterogeneity.

Our findings suggest that neural mobilisation interventions are mostly performed in patients where neurodynamic tests are thought to be positive (independent of criteria used). We only identified two studies in which neurodynamic tests were not used to define the study sample (Rezk-Allah et al. 2011; Waleed 2015). Unfortunately, both studies did not include a non-neural mobilisation control group, thereby preventing conclusions on the efficacy of neural mobilisations on patients with untested NM. However, even in a study population defined by neurological loss of function (electromyography, H-reflex) (Rezk-Allah et al. 2011), the neural mobilisation groups improved from baseline, suggesting that neurodynamic treatments are unlikely to make patients worse.

Neurodynamic tests can be negative in patients with clear nerve injury (Baselgia et al. 2017). Indeed, about a third of patients with ‘sciatica’ have negative SLR (Konstantinou et al. 2015; Mathieson et al. 2017). Despite this relatively large subgroup, we did not identify a single study that performed neural mobilisation interventions in patients with negative neurodynamic tests. Preclinical studies suggest that neurodynamic treatments may not only decrease neuropathic pain behaviour (Santos et al. 2012; Zhu et al. 2018), but also improve regeneration and remyelination (Da Silva et al. 2015; Martins et al. 2011), modulate biomarkers of inflammation (Giardini et al. 2017; Martins et al. 2011; Santos et al. 2012; Zhu et al. 2018) and opioid pathways (Martins et al. 2012; Santos et al. 2014). As such, preclinical benefits of neurodynamic interventions extend well beyond improving NM. Future studies will have to determine whether neurodynamic interventions are also beneficial in patients with nerve injury but without heightened nerve mechanosensitivity.

### Limitations

Whereas the study selection in the original search was undertaken by two investigators, the new search and article selection were performed by a single investigator. Most studies showed high risk of bias in one or more domains. In addition, most studies included relatively small sample sizes (range *n* = 11 to *n* = 56 in each group) and short duration of follow-up (maximum 2 months). The overall number of included studies was small, particularly in the NM_definite_ subgroup, and high heterogeneity was present, particularly in the NM_unclear_ group. This heterogeneity may, amongst other causes, be attributed to the varying neural mobilisation techniques and dosages used in different studies. Furthermore, the limited or unclear reporting of the criteria used to interpret the outcome of neurodynamic tests challenged a clear allocation of several studies in our systematic review. Of the 16 studies allocated to the NM_unclear_ subgroup, three did not specify the criteria used during neurodynamic testing (Dwornik et al. 2009; Kirthika et al. 2016; Lee & Kim 2017). Thus, some studies may have been wrongly attributed to the NM_unclear_ group. It is however unlikely that this has influenced our conclusions, because we found efficacy of neural mobilisation interventions independent of test criteria. Our findings might have additionally been influenced by other variations in diagnostic criteria used for spinally referred leg pain (e.g., use of neurological examination, MRI; see Online Appendix 1, Table 1). Critically, our findings highlight the importance of more careful reporting of criteria used for neurodynamic testing in future studies and the need for uniformly accepted criteria for neurodynamic testing and spinally related leg pain.

## Conclusion

Our review was unable to answer the question whether neural mobilisations are effective in patients with spinally referred leg pain and negative neurodynamic tests. However, we have shown a benefit of neural mobilisation for pain and disability in patients with NM independent of the criteria used during neurodynamic testing. Whereas firm conclusions are prevented by high risk of bias, small sample sizes and high heterogeneity across studies, our results currently do not support the view that the type of criteria used to define NM may majorly impact on the efficacy of neural mobilisation interventions.
